# Delay in the diagnosis of breast cancer during coronavirus pandemic

**DOI:** 10.17179/excli2020-3318

**Published:** 2021-01-20

**Authors:** Zohre Momenimovahed, Hamid Salehiniya

**Affiliations:** 1 Qom University of Medical Sciences, Qom, Iran; 2 Social Determinants of Health Research Center, Birjand University of MedicalSciences, Birjand, Iran

## ⁯

***Dear Editor,***

Since the beginning of coronavirus pandemic, numerous changes in the control, diagnosis, referral, treatment, and care of breast cancer have taken place all around the world. Immediately after the beginning of COVID-19 pandemic, strategies such as social distancing and home quarantine were introduced by health care systems to prevent the spread of coronavirus infection (Andersen, 2020[2]). Screening programs were also reduced to a great extent and only people with a registered illness were allowed to continue their treatment (de Pelsemaeker et al., 2021[6]). As the number of patient admission to hospital increased due to COVID-19, the rate of breast cancer screening reduced to a great extent due to the fear of coronavirus infection (Kaufman et al., 2020[8]). Also, as the pandemic intensified, health systems became heavily involved in the management of COVID patients in a way that, in many countries, health care providers began to postpone screening programs such as mammography and clinical breast examination in order to enable their staff to focus on COVID patients (Dietz et al., 2020[7]; Vanni et al., 2020[13]). On the other hand, most of the resources were focused on the COVID patients, strongly affecting other parts of the health system.

Breast cancer is the most common cancer in women (24.2 %), and is one of the leading causes of death (15 %) among them (Bray et al., 2018[3]). Over the past three decades, the number of breast cancer diagnosis in the world has doubled (Altobelli et al., 2017[1]). Early detection is associated with 5-year survival of localized and metastatic cancers (99 % vs. 28 %), (Carethers et al., 2020[4]). Also, 65-70 % of breast cancer cases are diagnosed by radiography measures and only 30-35 % of them are detected by physical examination. Despite the diagnostic efficiency of mammography, only emergency mammography is available during the coronavirus pandemic (Cedolini et al., 2014[5]; Puliti et al., 2017[12]), and this is because radiology centers are heavily involved in the diagnosis of COVID-19 and cannot respond to both coronavirus crisis and breast cancer screening. Although it is difficult to determine the effect of delayed diagnosis on the prognosis of breast cancer, we can be certain that the longer this time is, the greater its effect would be, and if no changes are made in the current situation, breast cancer is expected to be diagnosed at higher stages, which leads to poor prognosis that intimately, affects the capacity of health care system to manage breast cancer patients (Momenimovahed et al., 2020[10]). 

During the coronavirus pandemic, people with poor socioeconomic status are more affected by coronavirus and are more likely to lose their lives than others due to delayed cancer diagnosis (Printz, 2020[11]). Unfortunately, the rate of breast cancer screening is low among women in the world, so that the related disability-adjusted life-years (DALYs) in 2017 were 17,708,600 (Li et al., 2019[9]). The low rate of screening has caused a number of breast cancer patients to progress to advanced stages of cancer, causing some of them to require extensive treatment (Momenimovahed et al., 2020[10]). This problem in the use of screening programs has been worsened during the COVID-19 pandemic (Kaufman et al., 2020[8]). Therefore, policymakers should make every effort to minimize the effects of COVID-19 pandemic on breast cancer screening by taking into account the situation in their country. It seems that, one of the main measures that can be taken in this regard is to use private screening centers that are based outside the hospitals to minimize the concern of people regarding coronavirus infection. Also, tele-health programs that increase women's awareness and knowledge about breast self-examination should be promoted, so that suspicious cases can be identified in earlier time. For this purpose, the use of telephone counseling and triage of high-risk people through health records can be considered as effective measures. Finally, reducing the cost of screening programs while observing all health considerations, and increasing the working hours of health centers to reduce congestion can also be considered to reduce the burden of coronavirus on breast cancer screening programs.

## Conflict of interest

The authors declare no conflict of interest.
